# Supplementary material for: Advances in the Measurement of Polymeric Colorimetric Sensors Using Portable Instrumentation: Testing the Light Influence
Source: Polymers (Basel). 2022 Oct 12;14(20):4285. doi: 10.3390/polym14204285 (PMC9610941; doi:10.3390/polym14204285)
Supplement: Supplementary file 1 [file polymers-14-04285-s001.zip › polymers-1945825-supplementary.pdf]

# Advances in the measurement of polymeric colorimetric sensors using portable instrumentation: Testing the light influence

**A. Martínez-Aviño, M. de Diego- Llorente-Luque, C. Molins-Legua*, P. Campíns-Falcó ***

MINTOTA research group. Departament de Química Analítica, Facultat de Química,

Universitat de València, Dr. Moliner 50, 46100-Burjassot, Valencia, Spain.

***Corresponding authors: carmen.molins@uv.es; pilar.campins@uv.es**

**Supporting information**

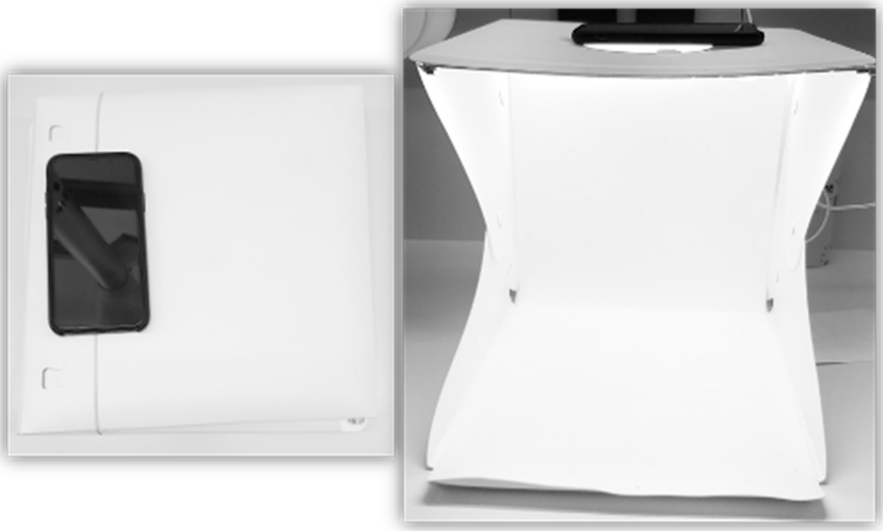

**Figure S1.** White box used for testing studied lights

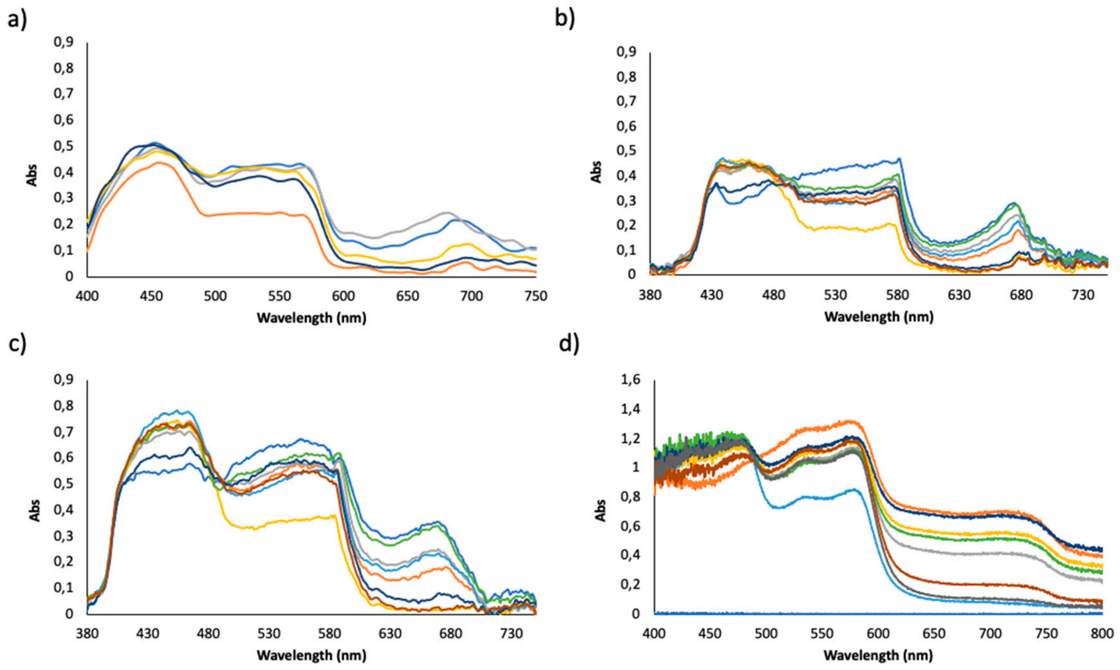


**Figure S2.** Spectra of the Red color group by using different lights a) Halogen lamp b) LED light c) Daylight and the mini-spectrometer compared to d) Laboratory benchtop spectrophotometer.

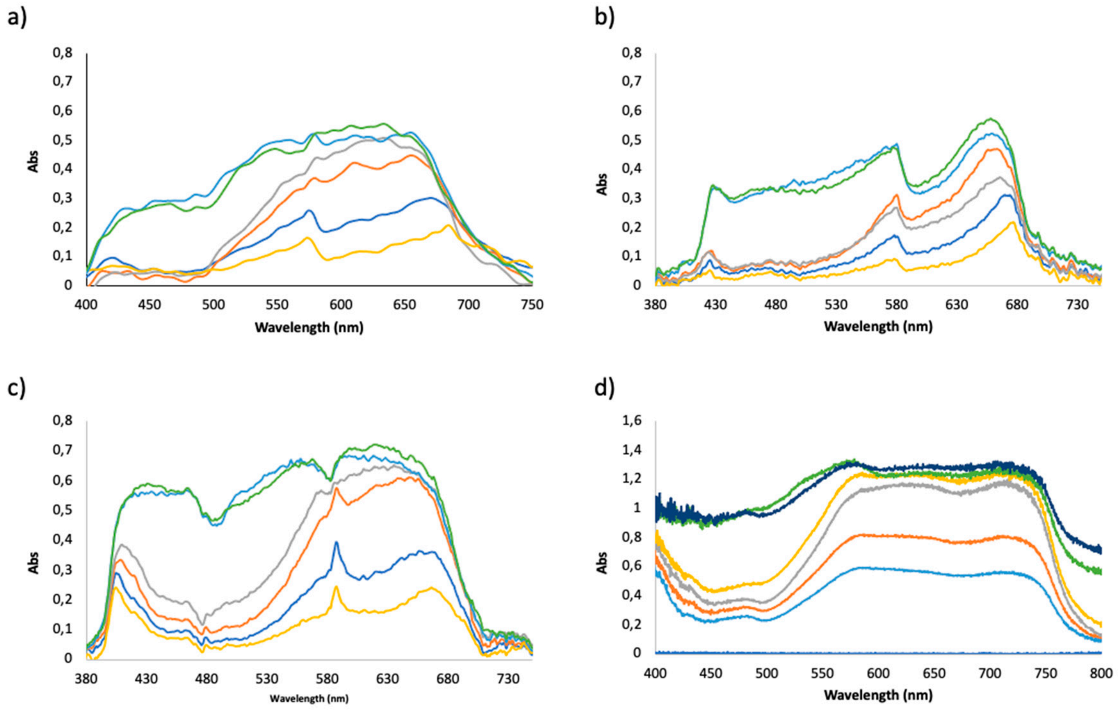


**Figure S3.** Spectra of the Blue color group by using different lights a) Halogen lamp b) LED light c) Daylight with the mini-spectrometer compared to d) Laboratory benchtop spectrophotometer.

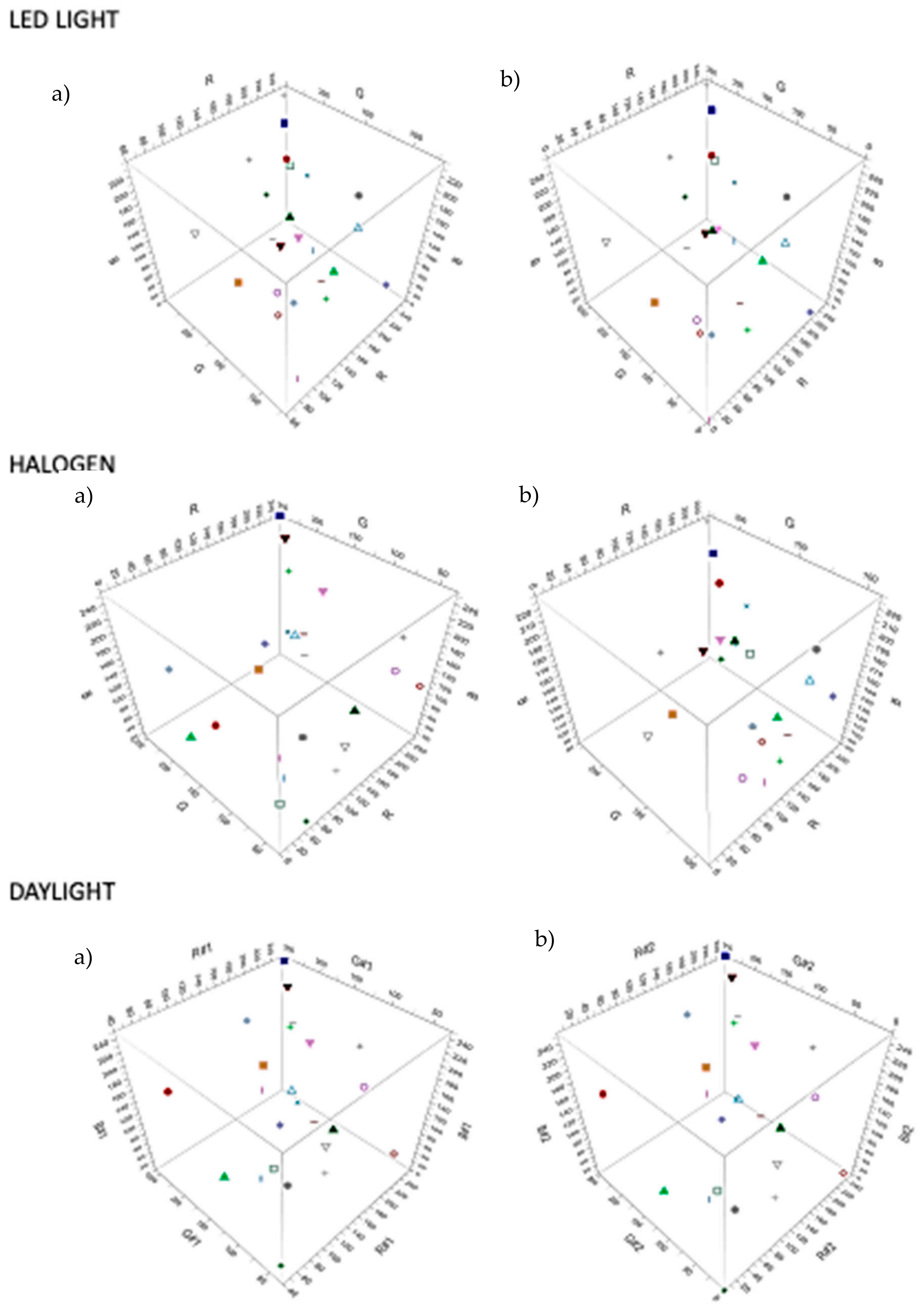


**Figure S4.** 3D distribution of the 24 colors of the correction palette according to their RGB parameters using different light sources. a) Unprocessed b) Processed
